# Mucosal and systemic T cell response in mice intragastrically infected with *Neospora caninum* tachyzoites

**DOI:** 10.1186/1297-9716-44-69

**Published:** 2013-08-10

**Authors:** Alexandra Correia, Pedro Ferreirinha, Amanda A Costa, Joana Dias, Joana Melo, Rita Costa, Adília Ribeiro, Augusto Faustino, Luzia Teixeira, António Rocha, Manuel Vilanova

**Affiliations:** 1 Laboratório de Imunologia Mário Arala Chaves, Departamento de Imuno-Fisiologia e Farmacologia, ICBAS-UP, Instituto de Ciências Biomédicas de Abel Salazar – Universidade do Porto, Rua de Jorge Viterbo Ferreira n° 228, Porto, 4050-313, Portugal; 2 IBMC - Instituto de Biologia Molecular e Celular, Porto, Portugal; 3 Departamento de Patologia e Imunologia Molecular, ICBAS-UP, Instituto de Ciências Biomédicas de Abel Salazar – Universidade do Porto, Rua de Jorge Viterbo Ferreira n° 228, Porto 4050-313, Portugal; 4 Departamento de Anatomia, ICBAS-UP, Instituto de Ciências Biomédicas de Abel Salazar – Universidade do Porto, Rua de Jorge Viterbo Ferreira n° 228, Porto 4050-313, Portugal; 5 UMIB-Unidade Multidisciplinar de Investigação Biomédica, Porto, Portugal

## Abstract

The murine model has been widely used to study the host immune response to *Neospora caninum*. However, in most studies, the intraperitoneal route was preferentially used to establish infection. Here, C57BL/6 mice were infected with *N. caninum* tachyzoites by the intragastric route, as it more closely resembles the natural route of infection through the gastrointestinal tract. The elicited T-cell mediated immune response was evaluated in the intestinal epithelium and mesenteric lymph nodes (MLN). Early upon the parasitic challenge, IL-12 production by conventional and plasmacytoid dendritic cells was increased in MLN. Accordingly, increased proportions and numbers of TCRαβ^+^CD8^+^IFN-γ^+^ lymphocytes were detected, not only in the intestinal epithelium and MLN, but also in the spleen of the infected mice. In this organ, IFN-γ-producing TCRαβ^+^CD4^+^ T cells were also found to increase in the infected mice, however later than CD8^+^ T cells. Interestingly, splenic and MLN CD4^+^CD25^+^ T cells sorted from infected mice presented a suppressive activity on in vitro T cell proliferation and cytokine production above that of control counterparts. These results altogether indicate that, by producing IFN-γ, TCRαβ^+^CD8^+^ cells contribute for local and systemic host protection in the earliest days upon infection established through the gastrointestinal tract. Nevertheless, they also provide substantial evidence for a parasite-driven reinforcement of T regulatory cell function which may contribute for parasite persistence in the host and might represent an additional barrier to overcome towards effective vaccination.

## Introduction

*Neospora caninum* is a protozoan parasite found in a wide range of domestic and wild animal hosts [1], and is responsible for clinical infections in dogs and cattle [2], having a major impact in dairy and beef industry [3]. Experimentally, the murine model has been the one preferred to study neosporosis, as it presented similar features to the infection occurring naturally in permissive hosts such as brain lesions [4], reproductive loss [5] and mother to fetus parasite transmission [6]. Although *N. caninum* is transplacentally transmitted in cattle with high efficiency, significant postnatal transmission also occurs in these animals [1], likely through oocyst ingestion [7]. Even though neosporosis can thus be established through the gastrointestinal (GI) tract, most studies on the host immune response have been carried out in hosts infected via the intraperitoneal (i.p.) or subcutaneous routes. Consequently, the mucosal immune response to this parasite in infected hosts was barely studied. As mucosal immunizations have been already attempted in experimental models of neosporosis [8-10], the characterization of the immune response to *N. caninum* in the mucosa and associated lymphoid tissues will be helpful to further understand the immunobiology of this parasitic disease. Therefore, a murine model of neosporosis established by intragastric (i.g.) administration of *N. caninum* tachyzoites was used here to study the immune response elicited by this parasite in the gut and associated lymphoid tissue of the infected hosts.

## Materials and methods

### Mice

Female C57BL/6 mice, 8–10 weeks old, were purchased from Charles River (Barcelona, Spain) and kept under specific pathogen-free conditions at the Animal Facility of Instituto de Ciências Biomédicas Abel Salazar (ICBAS), Porto, Portugal. Female p40^−/−^ C57BL/6 mice, 7–11 weeks old, were purchased from Jackson Laboratories (Bar Harbor, Maine, USA) and housed and bred also at ICBAS in individual ventilated cages. Nesting and housing material was provided as enrichment. All procedures involving mice were performed according to the European Convention for the Protection of Vertebrate Animals used for Experimental and other Scientific Purposes (ETS 123), 86/609/EEC Directive and Portuguese rules (DL 129/92). Authorization to perform the experiments was issued by the competent national board authority, Direcção Geral de Veterinária (0420/000/000/2008).

### Parasites

*Neospora caninum* tachyzoites (NC-1 isolate) were cultured and serially passaged in VERO cells maintained at 37 °C in Minimum Essential Medium (MEM) containing Earle’s salts (Gibco: Invitrogen Corporation, Carlsbad, CA, USA) supplemented with 10% fetal bovine serum (FBS), L-glutamine (2 mM), penicillin (200 IU/mL) and streptomycin (200 μg/mL) (all from Sigma, St Louis, USA) in a humidified atmosphere of 5% CO_2_ in air. Free parasitic forms of *N. caninum* were obtained as previously described [11] with slight modifications. Infected VERO cells were cultured until the host cell monolayer was 90% destroyed. Culture supernatants and adherent cells, harvested using a cell scraper, were centrifuged at 1500 × *g* for 15 min. The pellet was passed through a 25G needle and then washed three times in Phosphate Buffered Saline (PBS). The obtained pellet was suspended in 3 mL of PBS and passed through a PD-10 column filled with Sephadex™ G-25 M (Amersham Biosciences Europe GmbH, Freiburg, Germany). Parasite concentration was determined with a haemocytometer.

### Challenge infections

*N. caninum* infections in C57BL/6 mice were performed by the i.g. route using a previously described protocol [11]. Briefly, 5 h before infection mice were deprived of food. Mice were then anaesthetized by intramuscular injection of 20 μL of a 4:5 mixture containing xylazine (Rompum^®^, Bayer Portugal, S.A., Carnaxide) and ketamine (Imalgéne 1000, Bayer Portugal, S.A., Carnaxide). Stomach acidity was neutralized by directly administering into the stomach, with a gavage feeding needle linked to a 1-mL syringe, 50 μL of a 10% sodium bicarbonate solution in water. The same procedure was used to inoculate *N. caninum* tachyzoites 15 min later. Mice were i.g. challenged with 5 × 10^7^ tachyzoites in 0.2 mL of PBS or similarly inoculated with 0.2 mL of PBS and sacrificed at 6 h, 12 h, 18 h, 48 h, and 4, 7 and 21 days after challenge.

### Sample collection

At the different time points, mice were sacrificed upon isoflurane anesthesia by cervical dislocation. Spleens and mesenteric lymph nodes (MLN), from infected mice and non-infected controls, were aseptically removed and homogenized to single cell suspensions in HBSS for their usage in cell culture experiments and flow cytometry analysis. Additionally, brain, liver, MLN, and intestinal tissue samples were collected and either frozen (DNA isolation) or formalin-fixed (histology and immunohistochemistry). Small intestines were alternatively collected for IEL isolation. The number of animals per group per experiment is indicated in the respective figure legends.

### Histopathology and immunohistochemistry

Histopathology of intestinal tissue samples was assessed in formalin-fixed, paraffin-embedded 4 μm sections, mounted on amino-propyl-tri-ethoxy-silane (Sigma-Aldrich, St Louis, MO, USA) coated slides, of the gastrointestinal tract of mice 6, 12 and 18 h upon i.g. infection, stained with haematoxylin-eosin. The presence of *N. caninum* parasitic forms was assessed in similar tissue sections by immunohistochemistry, performed as previously described with slight changes [11]. Tissue sections were deparaffinized in xylene, rehydrated by graded washes of ethanol in water, ending in a final rinse in deionized water. Antigen retrieval was performed by incubating the slides in 10 mM citrate buffer (pH = 6) for 3 min in a pressure cooker. The slides were cooled and rinsed three times in Tris-buffered saline (TBS; 50 mM Tris, 150 mM NaCl, 0.1% Tween 20, pH = 7.6) for 5 min. Endogenous peroxidase activity was blocked by immersing slides in methanol containing 3% hydrogen peroxide for 10 min, followed by TBS washing. To reduce non-specific antibody binding, slides were incubated with normal rabbit serum (Dako, Glostrup, Denmark) diluted at 1:5 in TBS containing 10% bovine serum albumin (BSA), in a humidified chamber for 20 min at room temperature. Excess normal serum was removed and replaced by the goat anti-*N. caninum* antiserum (VMRD, Pullman, WA, USA) diluted at 1:2000. After one hour incubation at room temperature, slides were washed with TBS and incubated for 30 min with a 1:1000 dilution of peroxidase-labeled rabbit anti-goat secondary antibody (Millipore, Billerica, MA, USA). Slides were then washed with TBS and detection was performed for 3 to 5 min with 0.05% 3,3 diaminobenzidinetetrahydrochloride (DAB) freshly prepared in 0.05 M Tris/hydroxymethylaminomethane buffer, pH 7.6, containing 0.1% hydrogen peroxide (Dako). Finally, sections were lightly counterstained with Mayer’s haematoxylin, dehydrated and mounted in Entellan^®^ mounting medium (Merck, Darmstadt, Germany). Dilution of primary antibody and peroxidase-labeled secondary antibody were made with TBS containing 5% BSA. Positive control sections of *N. caninum*-infected IL-12^−/−^ mouse organs were included. Negative controls were performed by omitting the primary antibody incubation. Slides were evaluated under light microscopy.

### Real-time PCR analysis

DNA from intestinal tissue sections, MLN, liver and brain was isolated as previously described [11]. Detection of *N. caninum* DNA in infected tissue samples was assessed by a quantitative real-time PCR (qRT-PCR) analysis performed in a Corbett rotor gene 6000 system (Corbett life science, Sydney, Australia), using Express Sybr green ER qPCR supermix universal (Invitrogen, Carlsbad, CA, USA), for the amplification of a 337 bp sequence of the Nc5 region of *N. caninum* genome using the primers Np21plus 5’ CCCAGTGCGTCCAATCCTGTAAC 3’ and Np6plus 5’ CTCGCCAGTCAACCTACGTCTTCT 3’ (TIB-Molbiol, Berlin, Germany), both at a final concentration of 0.5 μM. The DNA samples were amplified using the following program: 95 °C for 10 min, followed by 45 cycles of 95 °C for 30 s, 63 °C for 20 s, and 72 °C for 45 s with fluorescence acquisition. A melting curve was performed in each run in order to confirm specificity of the amplicon: from 65 °C to 95 °C, with increments of 1 °C for 5 s. Parasite quantification was determined by interpolation of a standard curve, ranging from 10 to 10^-4^ ng of DNA extracted from *N. caninum* tachyzoites included in each run. Data were analyzed in the Rotor gene 6000 software v1.7 (Corbett life science).

### Intraepithelial lymphocyte isolation

Gut intraepithelial lymphocytes (IELs) were isolated as previously described [12]. Briefly, mice small intestines were removed and flushed with 20 mL of cold CMF (Ca^++^, Mg^++^ free Hank’s Balanced Salt Solution (HBSS) with 1 mM HEPES and 2% FBS, all from Sigma) using a syringe and needle. The Peyer’s Patches, fat and remaining mucous were removed along the intestine. The intestine was opened lengthwise, cut into 5 mm pieces and placed in a conical tube with 40 mL CMF. The pieces of tissue were washed twice with CMF by inverting the tube 10 times and letting the pieces settle before removing the supernatant. The intestine pieces were incubated in 25 mL CMF/DTE (CMF with 10% FBS and 1 mM dithioerythritol) (Sigma) at 37 °C and 100 rpm in an orbital incubator (GFL 3031, GFL, Burgwedel, Germany) for 20 min. The tube was vortexed at maximum speed for 15 s and the supernatant removed to a new tube. 25 mL of CMF/DTE were added to the tube containing the pieces of tissue and the vortexing step and collection of supernatant were done once more. All the incubation and supernatant collection steps were repeated. Supernatants from each intestine were pooled and centrifuged at 400 × *g*, 4 °C for 20 min. The pellet was suspended in 5 mL HBSS with 2% FBS and passed through a nylon wool column pre-wet with HBSS with 2% FBS (0.15 g teased nylon wool in a 5 cc syringe) and the column was washed with 20 mL HBSS with 2% FBS. The collected cell suspension was centrifuged and suspended in 16 mL of 44% Percoll™ (GE Healthcare Bio-Sciences AB, Uppsala, Sweden), adding 8 mL per 14 mL polystyrene round bottom tube. 5 mL of 67% Percoll were underlaid per tube and the 44%/67% Percoll gradients were centrifuged for 20 min at room temperature, 1400 × *g*, with the brake off. Cells from the interface were carefully removed with a Pasteur pipette and washed twice with 40 mL cold complete RPMI (RPMI 1640 supplemented with 50 U/mL penicillin, 50 μg/mL streptomycin, 1% HEPES buffer, 10% FCS and 5 μM 2-mercaptoethanol, all from Sigma). Pellet was suspended in complete RPMI.

### Flow cytometric analysis

The assessment of cell surface and cytoplasmic lineage or activation markers on different splenic leukocyte populations was performed by flow cytometric analysis (FACS). From spleen and MLN cell suspensions, prepared as described above, a number of 1 × 10^6^ leucocytes were stained per sample. The following monoclonal antibodies (mAbs), along with the respective isotype controls were used (at previously determined optimal dilutions) for immunofluorescence cytometric data acquisition in a Coulter EPICS XL flow cytometer (Beckman Coulter, Inc., Brea, CA, USA): fluorescein isothiocyanate (FITC) anti-mouse/rat Foxp3 (FJK-16 s), phycoerythrin (PE) anti-mouse TCR β (H57-597) and PE-Cy5 rat anti-mouse CD4 (L3T4) (RM4-5) (all from eBioscience, San Diego, CA, USA); Biotin anti-mouse PDCA-1 (JF05-1C2.4.1) (Miltenyi Biotech, Inc. Auburn, CA, USA); FITC hamster anti-mouse CD11c (HL3), PE-Cy5 rat anti-mouse CD8a (53–6.7), PE anti-mouse CD25 (PC61), PE rat anti-mouse IL-4 (BVD4-1D11), Biotin hamster anti-mouse γδ T-cell receptor (GL3), FITC anti-mouse IFN-γ (XMG1.2), FITC rat anti-mouse IL-17A (TC11-18H10), PE rat anti-mouse IL-10 (JES5-2A5) (all from BD Pharmingen, San Diego, CA, USA). Biotin conjugated mAbs were revealed with Streptavidin-PE-Cy7 (BD Pharmingen). Cells were preincubated for 15 min with anti-FcγR (a kind gift of Dr Jocelyne Demengeot, Gulbenkian Institute of Science, Oeiras, Portugal) before CD11c and Foxp3 staining. The Foxp3 Staining Buffer Set (eBioscience) was used for fixation and permeabilization of splenocytes previously surface stained with CD4 and CD25 mAbs. Data were analysed by using CELLQUEST software (Becton-Dickinson, San Jose, CA, USA).

### Intracellular staining

The intracellular expression of the cytokine IFN-γ was detected in splenic and MLN CD8^+^ and CD4^+^ T lymphocytes, as well as in IELs. IL-4, IL-17A and IL-10 expression was also evaluated in splenic and MLN CD4^+^T cells. Splenocytes and MLN cells were obtained as described above. Red blood cell lysis was performed in spleen suspensions by incubation with 0.15 M ammonium chloride. Cells were washed and suspended in complete RPMI medium. Spleen, MLN or IEL 1 × 10^6^ cells were transferred to 96-well tissue culture plates (Nunc, Roskilde, Denmark) and stimulated for 4.5 h with 20 ng/mL phorbol myristate acetate and 200 ng/mL ionomycin in the presence of 10 ng/mL of brefeldin A (all from Sigma). Staining of cell surface markers CD4, CD8, TCR β and TCRγδ was performed as described above, after a preincubation step of 15 min with anti-FcγR, followed by fixation with 2% formaldehyde. Cells were permeabilized with 0.5% saponin in flow cytometric buffer (PBS containing 1% BSA and 0.01 M sodium azide) and, subsequently, cells were incubated for 15 min with anti-FcγR and stained for 30 min at room temperature with the appropriate antibody. The intracellular expression of the cytokines IL-12 and IL-10 was detected in splenic and MLN conventional and plasmacytoid dendritic cells (cDC and pDC, respectively). Spleen and MLN suspensions were enriched with DC by magnetic sorting using anti-CD11c beads (Miltenyi), according to the manufacturer’s instructions. 1 × 10^6^ cells were then placed in 96-well tissue culture plates (Nunc) and incubated for 4.5 h with 10 ng/mL of brefeldin A (Sigma). Cells were surface stained with PE-Cy5.5 hamster anti-mouse CD11c (HL3) (BD Pharmingen) and Biotin anti-mouse PDCA-1 (Miltenyi Biotech) revealed with PE-Cy7 conjugated streptavidin (BD Pharmingen). Intracellular staining was performed with PE rat anti-mouse IL-12 (p40/p70) (C15.6) and FITC rat anti-mouse IL-10 (JES5-2A5) (all from BD Pharmingen). Intracellular staining with the isotypic controls was performed to confirm the specificity of antibody binding.

### Cell cultures and suppression assays

For anti-CD3 mAb-stimulated cultures, antigen presenting cells (APC) were prepared from naïve splenic or MLN single cell suspensions by layering 5 mL onto 2.5 mL of a polysucrose-sodium ditrizoate solution (Histopaque 1083^®^, Sigma) and centrifuging at 800 × *g* for 20 min at room temperature. Mononuclear cells collected from the medium-Histopaque interface were washed, suspended in RPMI complete medium and irradiated at 3000 rad in a Gammacell 1000 Elite irradiator (Nordion International, Inc., Ottawa, Canada). Total CD4^+^ and the T cell subsets CD4^+^CD25^-^ and CD4^+^CD25^+^ from non-infected and infected mice were isolated from pooled spleen or MLN cells of five mice per group, by using a magnetic cell sorting CD4^+^CD25^+^ T-cell isolation kit (Miltenyi Biotech, Inc., Auburn, CA, USA) following the manufacturer’s instructions. Purity of the sorted cells routinely ranged between 92-98%. Naïve CD4^+^CD25^-^ T cells (responder cells) were plated at 2.5 × 10^4^/well in U-shape 96-well plates together with 10^5^ APC without stimulus or stimulated with 1 μg/mL anti-CD3 mAb (145.2C11) (BD Pharmingen). CD4^+^CD25^+^ T cells sorted from control and *N. caninum*-infected mice were added to the naïve CD4^+^CD25^-^ T cells in a 1:1 proportion. Each condition was set in sextuplicates and culture was maintained for 72 h. Supernatants from these cell cultures were collected and stored at −80 °C until further use. The CellTraceTM CFSE Cell Proliferation Kit (Molecular Probes, Invitrogen, Eugene, OR, USA) was used for cell labelling. A CFSE (5-(and-6)-carboxyfluorescein diacetate succinimidyl ester) stock solution (10 mM in DMSO) stored at −20 °C was thawed and diluted in PBS with 0.1% BSA to a final concentration of 10 μM. Naïve CD4^+^CD25^-^ T cells (responder cells) were suspended at 2 × 10^6^/mL in PBS with 0.1% BSA and further incubated with an equal volume of the diluted CFSE solution, for 7 min at room temperature. Excess CFSE was quenched by adding 1/5 of the volume of heat inactivated FBS. Cells were washed three times with complete RPMI medium. Responder cells were plated at 2.5 × 10^4^/well in U-shape 96-well plates together with 10^5^ APC and 1 μg/mL anti-CD3 mAb. In order to evaluate Treg cell suppressive function, CD4^+^CD25^+^ T cells from control and infected mice were added at different responder: CD4^+^CD25^+^ T cell ratios (1:1, 2:1, and 10:1). Responder cells without anti-CD3 stimulus were used as the negative control. Stimulated responder cells with no suppressor populations added were used as the positive control. Unlabelled stimulated responder cells were used to define cell auto fluorescence. Other controls consisted of stimulated responder cells co-cultured with CD4^+^CD25^-^ T cells from the different animal groups tested, to exclude suppression due to cell number/well. Each condition was set in sextuplicates and cultures were maintained for 72 h at 37 °C and 5% CO_2_. Proliferation/suppression was determined based on CFSE fluorescence by flow cytometric analysis.

For antigen stimulated cultures, bone marrow-derived dendritic cells (BMDC) were prepared by a granulocyte macrophage colony-stimulating factor (GM-CSF)-based method, as described by Lutz et al. [13]. Upon differentiation, BMDC were antigen-loaded by overnight incubation with 100 μg/mL of *N. caninum* sonicates prepared as previously described [11], or cultured without antigen in the presence of 50 ng/mL of lipopolysaccharide from *E. coli* (Sigma). BMDC APC were then washed twice with PBS and suspended in complete RPMI. CFSE-labelled responder cells (CD4^+^CD25^-^ cells isolated, as described above, from infected mice, 7 days upon infection) were plated at 2.5 × 10^4^/well in U-shape 96-well plates together with 10^5^ BMDC and were used as the positive control. To evaluate Treg suppression capacity, CD4^+^CD25^+^ T cells from control and infected mice were added to the cultures at 1:1 and 2:1 responder: CD4^+^CD25^+^ T cell ratios.

### IFN-γ, IL-4, and IL-10 measurements

The concentration of IFN-γ, IL-4 and IL-10 in cell culture supernatants from *N. caninum*-infected mice and from non-infected controls were quantified with the Mouse IFN-γ, IL-4 and IL-10 ELISA Ready-Set-Go!^®^ kits (eBioscience), according to manufacturer’s instructions.

### Statistical analysis

Unless otherwise indicated, statistical significance of results was determined by unpaired Student *t*-test, using the GraphPad Prism 4 Software (GraphPad Software, Inc., La Jolla, CA, USA). Results were considered statistically significant with *P* values of less than 0.05.

## Results

### Infection of C57BL/6 mice with *N. caninum* tachyzoites administered intragastrically

We have previously reported that neosporosis could be established in mice i.g. challenged with *N. caninum* tachyzoites. However, in the i.g.-challenged mice, the immune response elicited in the gut associated lymphoid tissue was studied only in the Peyer’s Patches [11]. Here, C57BL/6 mice were challenged i.g. with *N. caninum* tachyzoites to evaluate the elicited immune response in the gut epithelium and draining lymph nodes. Early after the i.g. challenge, parasitic DNA was detected by qRT-PCR in intestinal tissue samples (1/4, 2/4 and 1/4 mice at 6, 12 and 18 h, respectively) and MLN (1/4 mice at 6 h). The presence of tachyzoites within the intestinal tissue was confirmed by immunohistochemistry (Additional file 1). No evident signs of inflammation were observed in intestinal tissue samples of the infected mice up to 18 h upon infection, as evaluated by microscopic observation of haematoxilin/eosin stained paraffin sections (data not shown). In the infected mice, the presence of *N. caninum* DNA was assessed in the liver and brain at 4 and 7 days upon infection, in pooled samples of two independent experiments. Parasitic DNA was detected by qRT-PCR in both organs in 4/10 mice and 6/10 mice, 4 and 7 days after infection, respectively. Similarly infected C57BL/6 mice still presented parasitic DNA in the brain 21 days after infection (2/4) and 5/5 survived infection for at least six months. A more exhaustive analysis would nevertheless be necessary to determine whether the surviving mice were chronically infected. These results show that in C57BL/6 mice *N. caninum* tachyzoites can disseminate in the host from the GI tract. Results also show that the parasite i.g.-challenged mice control acute infection.

### MLN cDC and pDC produce IL-12 early upon i.g. challenge with *N. caninum* tachyzoites

**Figure 1:**
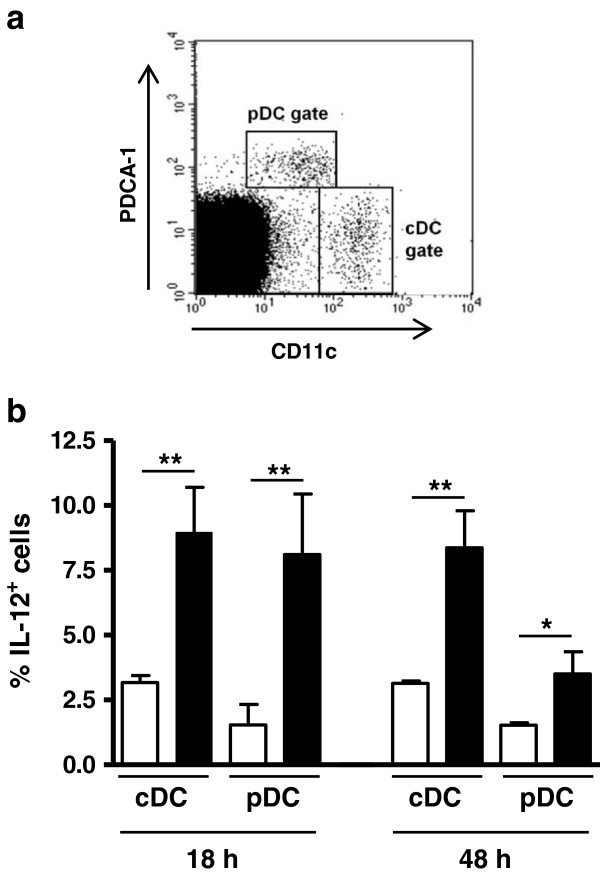
**cDC and pDC produce IL-12 in response to *N. caninum *i.g. infection. (a)** Representative example of flow cytometric analysis of surface PDCA-1 and CD11c expression on total MLN leukocyte cells. Dot plots represent cells collected from C57BL/6 mice 18 h after i.g. challenge with 5 × 10^7^*N. caninum* tachyzoites. Gates were set as shown to delineate cDC (CD11c^high^) and pDC (CD11c^low^ PDCA-1^high^). **(b)** Proportion of IL-12^+^ cells within cDC and pDC populations in the MLN of C57BL/6 mice, evaluated by flow cytometric analysis, at the specified time points after i.g. treatment with PBS (open bars) or i.g. inoculation with 5 × 10^7^ *N. caninum* tachyzoites (closed bars). Bars represent the mean plus one standard deviation of three animals in the PBS group and four animals in the infected mice group. This is one representative result of two independent experiments (*P<0.05; **P<0.01).

As we have shown in a previous report a large proportion of splenic conventional and plasmacytoid dendritic cells (cDC and pDC, respectively) produce IL-12 in BALB/c mice infected i.p. with *N. caninum* tachyzoites [14]. This cytokine is a crucial factor in mediating host immune protection against neosporosis [15-17]. As shown in Figure 1, an increased frequency of IL-12-expressing cDC and pDC was detected in the MLN of infected mice, 18 and 48 h upon the parasitic challenge. The frequency of IL-10-producing MLN cDC and pDC was also evaluated and did not significantly change upon infection (data not shown). These results show that *N. caninum* tachyzoites administered i.g. induce IL-12 production by host dendritic cells (DC) in the draining lymph nodes.

### Increased frequencies of TCRβ^+^CD8^+^IFN-γ^+^ IEL were observed in C57BL/6 mice challenged i.g. with *N. caninum* tachyzoites

**Figure 2:**
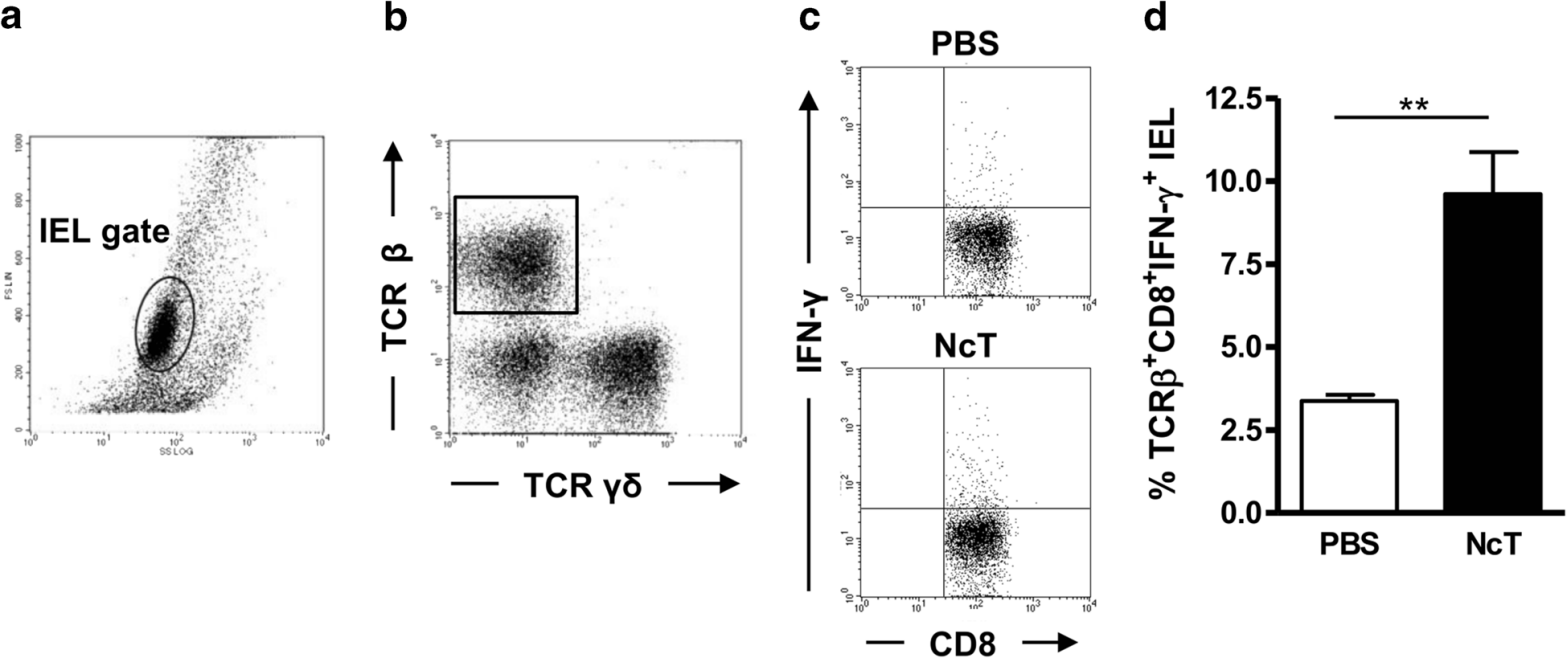
**Increased expression of IFN-γ by TCRβ**^**+**^**CD8**^**+ **^**IEL in *N. caninum *infected mice.** Representative dot plots of **(a)** cells isolated from the small intestines of C57BL/6 mice (IEL were gated as shown); **(b)** TCRγδ and TCRβ IEL. **(c)** Gated TCRβ CD8^+^ IEL expressing IFN-γ in control (PBS) and *N. caninum*-infected mice (NcT). **(d)** Frequency of TCRβ^+^CD8^+^ IFN-γ^+^ IEL in control and *N. caninum* i.g.-infected mice, 48 h after challenge. Bars represent mean plus one standard deviation of three animals in the PBS group and four animals in the infected mice group. This is one representative result of three independent experiments (*P<0.05).

Murine gut IEL comprise both αβ and γδ TCR^+^ cells [18] which have been shown to mediate host protection against enteric infections, including those caused by protozoans [19]. Here, an increased frequency of TCRβ^+^CD8^+^IFN-γ^+^ IEL was observed in C57BL/6 mice comparatively to mock-infected controls, 48 h upon i.g. challenge with *N. caninum* tachyzoites (Figure 2). Conversely, no differences were found between infected mice and controls in the frequencies of IFN-γ-producing TCRγδ^+^ (1,30 ± 0,27 vs 1,13 ± 0,29) or TCRβ^+^CD4^+^ (2,70 ± 0,82 vs 1,84 ± 0,76) IEL. Production of IL-17A by TCRγδ^+^ IEL was not detected either in controls or infected mice. These results indicate that in the gut, CD8^+^αβTCR^+^, but not γδTCR^+^, IEL are activated by i.g.-administered *N. caninum* tachyzoites and produce the host protective cytokine IFN-γ.

### CD8^+^ T cells produce IFN-γ in the MLN of *N. caninum* i.g.-infected mice

**Figure 3:**
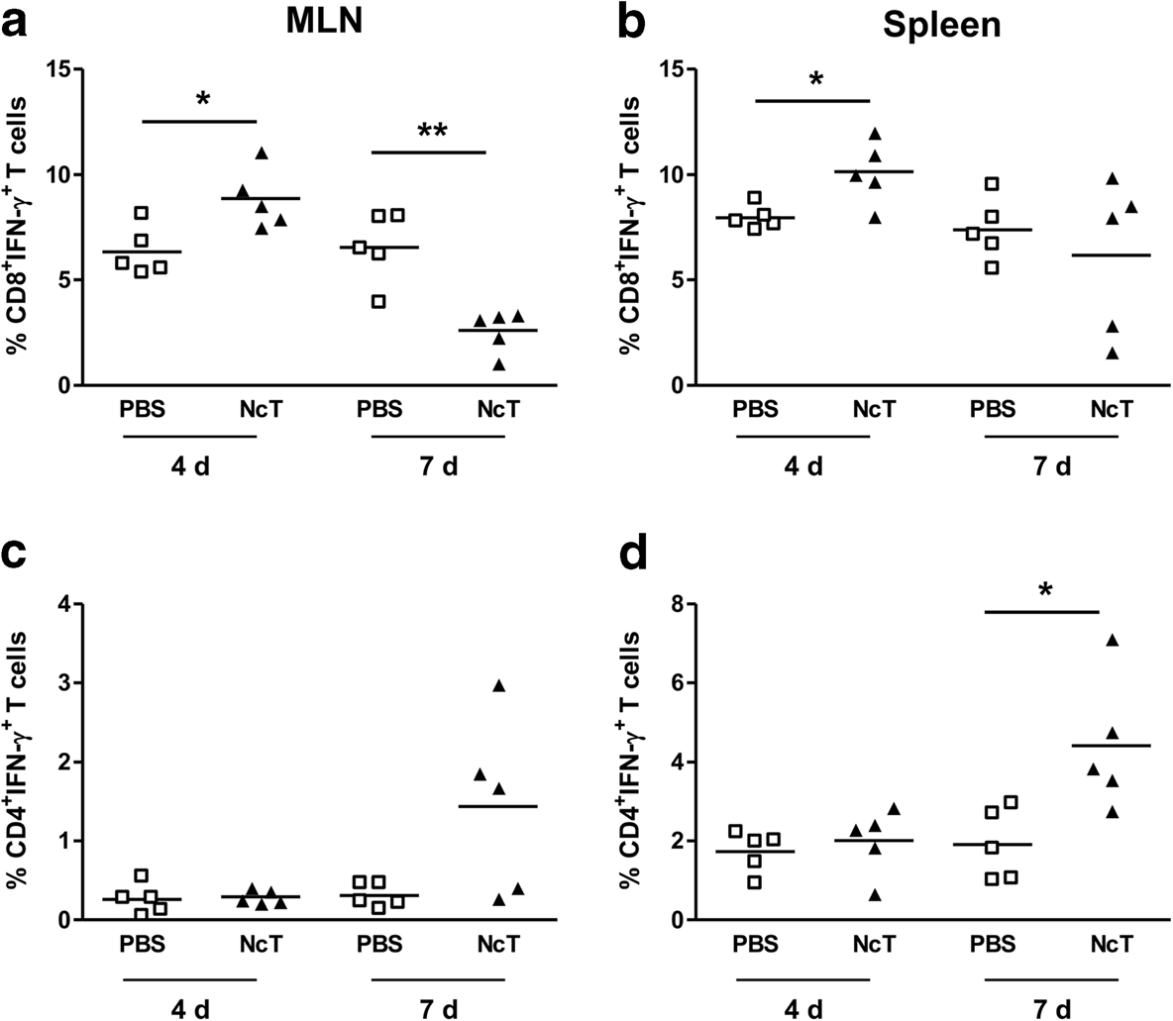
**Expression of IFN-γ in splenic and MLN CD8**^**+ **^**and CD4**^**+ **^**T cells.** Scatter plots of frequency of CD8^+^IFN-γ^+^ T cells **(a** and **b)** or CD4^+^IFN-γ^+^ T cells **(c** and **d)**, as indicated, in the MNL **(a** and **c)** and spleen **(b** and **d)** of control (PBS) or *N. caninum*-infected mice (NcT), 4 and 7 days upon i.g. challenge. Each symbol represents an individual mouse; horizontal bars correspond to mean values of the respective group. Five mice per group were used. This is one representative result of three independent experiments. Statistical significance between groups is indicated above symbols (*P<0.05; **P<0.01).

IL-12 drives the differentiation of T cells towards an IFN-γ-producing phenotype. As increased production of IL-12 was observed in the MLN of *N. caninum* infected mice, the frequency of CD4^+^ and CD8^+^ T cells producing IFN-γ was assessed therein, 4 and 7 days upon infection. As shown in Figure 3a, an increased frequency of CD8^+^IFN-γ^+^ T cells was observed in the MLN of infected mice. This increase was observed at day 4 post-infection whereas at day 7 it was found below control values. No such increase was observed for CD4^+^IFN-γ^+^ T cells at the assessed days (Figure 3c). Nevertheless, at day 7 upon infection some mice presented CD4^+^IFN-γ^+^ T cells at higher frequency than controls. This high variability was observed in the three experiments done. In order to determine whether IFN-γ production could also be induced in splenic T cells upon the i.g. infection, their frequency and number was similarly assessed. As shown in Figure 3b, increased proportions of CD8^+^IFN-γ^+^ T cells were also observed in the spleen 4 days upon infection that were found within control values by day 7. In contrast, the frequency of CD4^+^IFN-γ^+^ T cells, which was similar to controls 4 days after infection, significantly increased at day 7 (Figure 3d). The frequency of CD4^+^ T cells expressing IL-4, IL-10 or IL-17A was not different between controls and infected mice at both assessed time points and lymphoid tissues (data not shown). These results show that in the i.g. infected mice CD8^+^ T cells are early producers of IFN-γ, not only at the intestinal mucosa and draining lymph nodes but also systemically, as detected in the spleen.

### Increased suppressive activity of CD4^+^CD25^+^ T cells from infected mice

**Figure 4:**
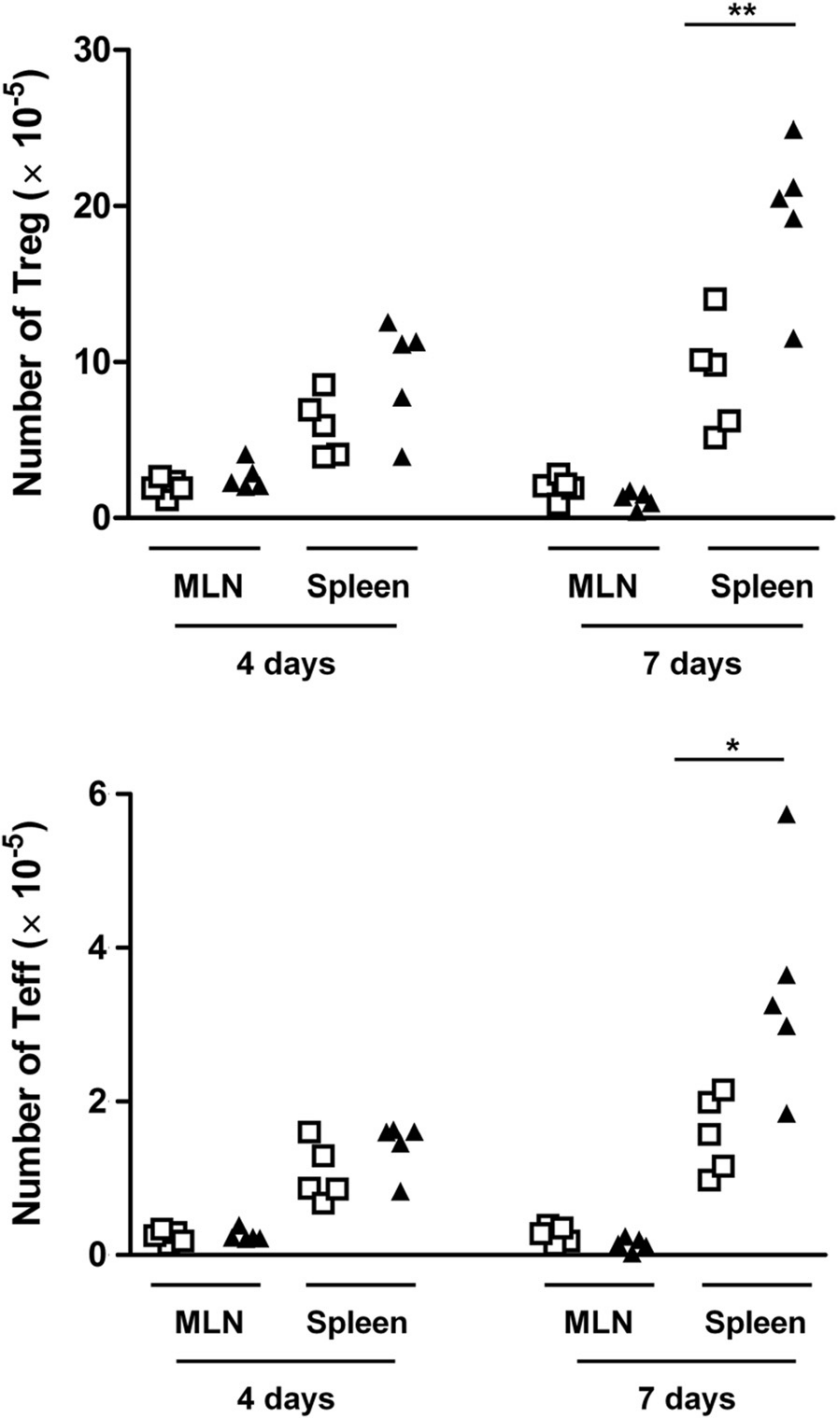
**Spleen and MLN Treg and Teff cell numbers.** Scatter plots of CD4^+^CD25^+^ Treg (Foxp3^+^) and Teff (Foxp3^-^) cell numbers in the MLN and spleen of control (open squares) or *N. caninum*-infected mice (closed triangles), 4 and 7 days upon the i.g. challenge. Each symbol represents an individual mouse; horizontal bars correspond to mean values of the respective group. Five mice per group were used. This is one representative result of three independent experiments. Statistical significance between groups is indicated above symbols (*P<0.05; **P<0.01).

**Figure 5:**
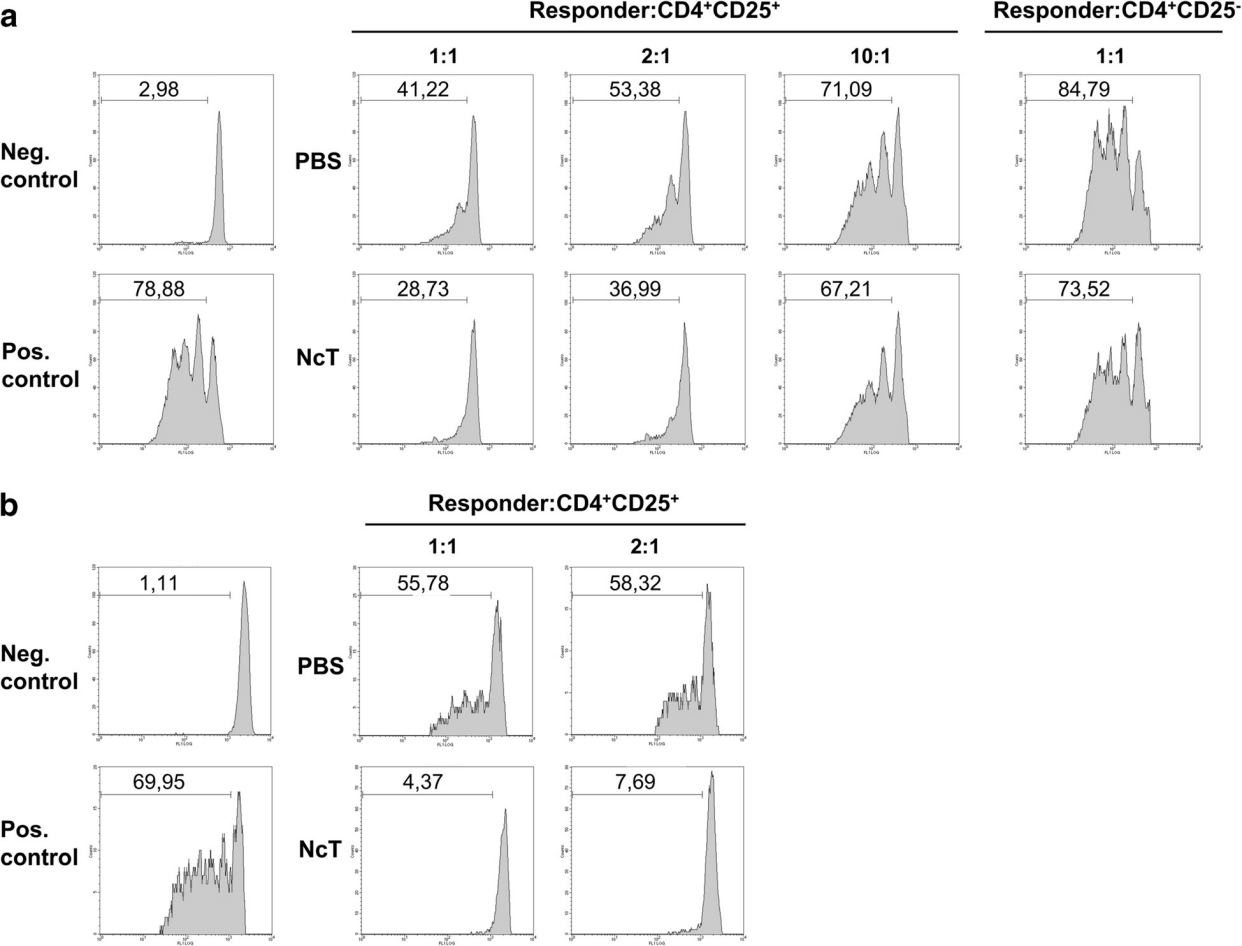
**High suppressive activity of Treg from *N. caninum*-infected mice. (a)** Flow cytometric evaluation of anti-CD3 mAb (1 μg/mL) induced proliferative response of 2.5 × 10^4^ CFSE-labelled naïve CD4^+^CD25^-^ T (responder) cells cultured for 72 h with 10^5^ irradiated APC/well, in the absence (Pos. control) or presence of CD4^+^CD25^+^ T cells obtained from control mice or mice infected with *N. caninum*. Neg. control corresponds to responder cells with no mAb added. Histograms correspond to 1:1, 2:1 and 10:1 responder: CD4^+^CD25^+^ cell ratios or 1:1 responder: CD4^+^CD25^-^ T cell ratio, as indicated. **(b)** Flow cytometric evaluation of *N. caninum* antigen-induced proliferative response of 2.5 × 10^4^ CFSE-labelled splenic CD4^+^CD25^-^ T (responder) cells obtained from 7 day-infected mice, cultured for 72 h with 10^5^ antigen-loaded BMDC/well, in the absence (Pos. control) or presence of CD4^+^CD25^+^ T cells obtained from control mice or mice infected with *N. caninum*. Non-specific proliferation control (Neg. control) corresponds to responder cells cultured with LPS (50 ng/mL) activated BMDC, without *N. caninum* antigen. Histograms correspond to 1:1 and 2:1 responder: CD4^+^CD25^+^ cell ratios, as indicated. Numbers within histograms correspond to the percentages of cells that divided at least once. CD4^+^CD25^+^ cells added in each condition were sorted from pooled splenic cells of 5 mice per group. Results are a representative example out of three **(a)** or one out of two **(b)** independent experiments.

We have previously reported that in *N. caninum* i.g.-infected BALB/c mice, higher numbers of CD4^+^CD25^+^Foxp3^+^ cells (T regulatory cells, Treg), as well as of CD4^+^CD25^+^Foxp3^-^ cells (T effector cells, Teff), were detected in the spleen by 8 days upon the parasitic challenge [11]. Accordingly, increased numbers of CD4^+^CD25^+^ cells were found in the C57BL/6 infected mice at day 7, but not at day 4 upon infection. As *N. caninum* i.g. infection did not significantly change the relative proportions of Treg and Teff (Additional file 2), the numbers of Treg and Teff are proportionally increased (Figure 4). Interestingly, 7 days after the parasitic challenge, the frequency of CD4^+^CD25^-^ cells expressing the Treg marker Foxp3 was increased in the spleen of infected mice (Additional file 2). No such differences were observed 4 days upon infection or at any assessed time point in the MLN (data not shown). To determine whether, regardless of similar Treg and Teff proportions, splenic CD4^+^CD25^+^ T cells sorted from *N. caninum*-infected and control mice could have dissimilar suppressive activity, a suppression assay was performed by co-culturing CD4^+^CD25^+^ T cells sorted from infected or control mice with CFSE-labelled naïve CD4^+^CD25^-^ T cell responders. Interestingly, CD4^+^CD25^+^ T cells obtained from infected mice suppressed more efficiently the anti-CD3 mAb-induced proliferation of T cell responders than did the control CD4^+^CD25^+^ counterparts. This effect was observed at any of the assessed responder: CD4^+^CD25^+^ T cell ratios (1:1; 2:1; 10:1). Curiously, when CD4^+^CD25^-^ T cells from *N. caninum*-infected mice were co-cultured with responder cells, a noticeable suppression of responders proliferation was also observed, which did not occur when CD4^+^CD25^-^ T cells from controls were used (Figure 5a). This might be a consequence of the increased frequency of Foxp3-expressing cells within this cell subset detected in the 7-day infected mice, which in mice are known to have a regulatory function [20]. In order to determine whether the observed disparate suppressive effect of Treg from infected and control mice could also occur in *N. caninum*-antigen stimulated cultures, antigen-loaded BMDC were used as APC to stimulate T cell proliferation/suppression. Activation of BMDC upon incubation with *N. caninum* antigen extracts was confirmed by up-regulated expression of surface MHC class II, CD40 and CD86, detected by using flow cytometry (data not shown). As shown in Figure 5b, antigen-driven T cell proliferation was also more effectively suppressed by Treg from infected mice than by Treg from non-infected controls.

**Figure 6:**
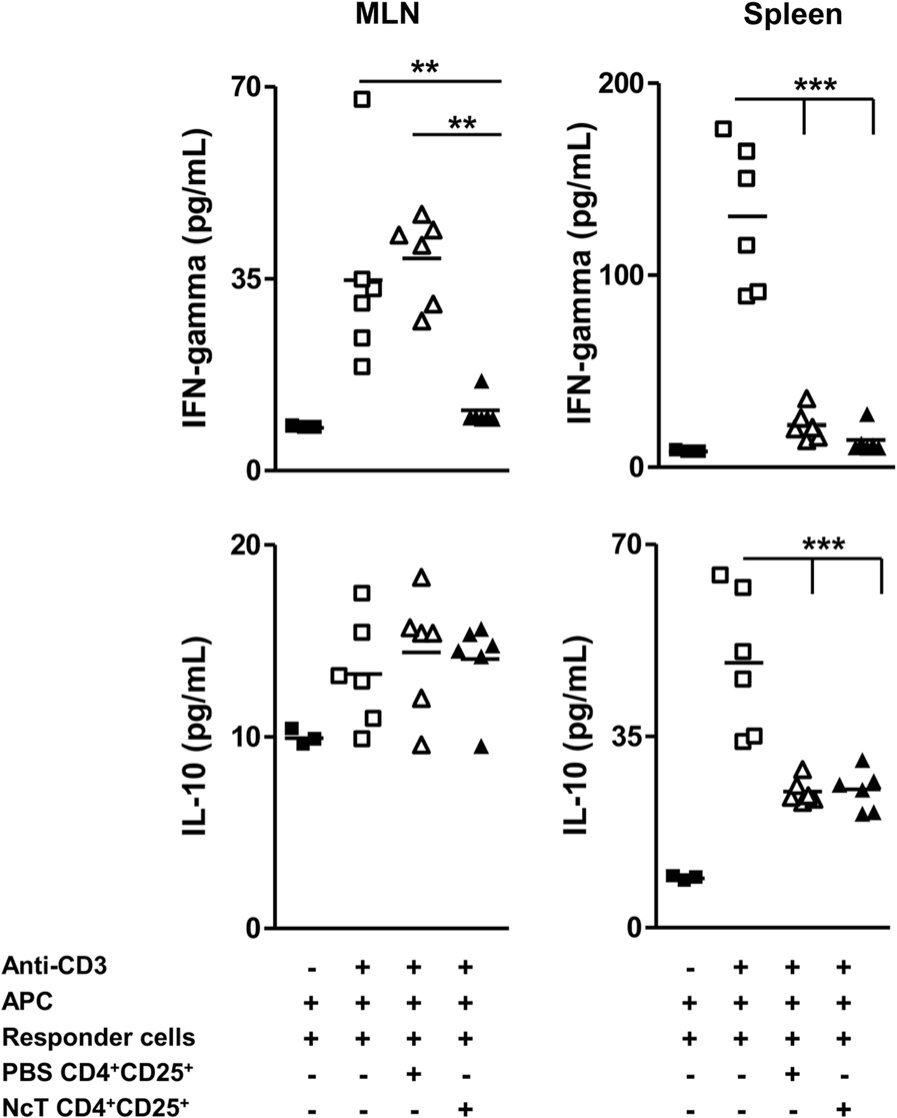
**Suppression of cytokine production by splenic and MLN Treg.** IFN-γ and IL-10 cytokine concentration in the supernatants of MLN or splenic naïve 2.5 × 10^4^ CD4^+^CD25^-^ T cells cultured for 72 h with anti-CD3 mAb (1 μg/mL) and 10^5^ APC, alone or in the presence of 1:1 CD4^+^CD25^+^ MLN or splenic T cells from control or *N. caninum*-infected mice, as indicated. Each symbol represents an individual culture well. Horizontal bars represent the mean values of the respective group. CD4^+^CD25^+^ cells added in each condition were sorted from pooled MLN or splenic cells of 5 mice per group, as indicated. Results are a representative example out of three independent experiments. Statistical significance among groups was determined by one-way ANOVA, followed by a Bonferroni post-test, and is indicated above symbols (**P<0.01; ***P<0.001).

Decreased levels of IFN-γ and also of IL-10 were detected in the supernatants of anti-CD3 mAb-stimulated co-cultures of splenic naïve CD4^+^CD25^-^ T cells when co-cultured with splenic CD4^+^CD25^+^ T cells from infected and control mice. CD4^+^CD25^+^ T cells from both *N. caninum*-infected mice and non-infected controls, efficiently suppressed the production of IFN-γ and IL-10 to levels similar to those of non-stimulated cells. The equivalent suppression of cytokine production by Treg from both groups, when a higher suppressive activity on T cell proliferation was observed for Treg from infected mice might result from the high Treg: T responder ratio (1:1) which may have prevented differences to show up. Interestingly, in similar co-cultures of cells sorted from the MLN, CD4^+^CD25^+^ T cells from the MLN of infected mice suppressed more efficiently the production of IFN-γ by MLN responder cells than CD4^+^CD25^+^ T cell counterparts from non-infected controls. In contrast, in the latter cultures, no suppression of IL-10 production was observed likely because IL-10 levels in the supernatants of stimulated cultures were no significantly different from the ones found in non-stimulated cultures (Figure 6). No differences were found in the levels of IL-4 in any of the conditions tested (data not shown). Altogether, these results show that CD4^+^CD25^+^ T cells from *N. caninum*-infected mice display an enhanced suppressive activity when compared with the equivalent T cell population from non-infected controls.

## Discussion

Although *N. caninum* can infect its natural hosts through the GI tract [7], very little is known about the local immune response in the intestinal mucosa and associated lymphoid tissue. We have previously reported that neosporosis could be established in mice i.g. challenged with *N. caninum* tachyzoites [11]. Although this parasitic form may present antigenic differences from oocysts and sporozoites, this model may nevertheless better mimic the natural infection route in horizontally transmitted neosporosis, than the intraperitoneal or subcutaneous routes. The i.g. infection model was used here to study the immune response elicited in the intestinal mucosa and MLN. In infected mice, tachyzoites could be detected within the intestinal tissue early after the parasitic challenge. Also soon after infection, parasitic DNA was detected in the MLN of one infected mouse. This might explain the stimulatory effect on MLN cDC and pDC observed in the infected mice, and their increased expression of IL-12, a key cytokine in mediating host immune protection against *N. caninum* infections [14-16]. On the other hand, as DC have been shown to help systemic dissemination of *N. caninum *[21] and of the closely-related protozoan *T. gondii *[22,23], it would be interesting to determine whether DC may transport the parasites from the gut to the MLN, contributing for parasite dissemination within the host. The detection of *N. caninum* DNA in the MLN of an i.g.-infected mouse as early as 6 h after infection might support this hypothesis, though further studies must be carried out to confirm such a role of DC in this infection model. As parasite DNA was detected in the brain of infected mice 21 days upon infection, this confirmed previous results showing that *N. caninum* tachyzoites might cross the intestinal epithelial barrier and disseminate to other organs [11]. Lack of evident signs of disease in the infected mice indicates that mice are able to control neosporosis established by the i.g. route. Nevertheless, it cannot be excluded that this control might in part be due to a low number of parasites successfully crossing the intestinal epithelial barrier.

Our results, by showing that MLN DC produce IL-12 in response to *N. caninum* infection are in agreement with our previous observation that both cDC and pDC produced IL-12 in the spleen of mice infected i.p. with *N. caninum* tachyzoites [14] and with other reports showing in vivo [15] and in vitro [24,25] production of this cytokine upon DC stimulation with this parasitic form. Both cDC and pDC populations were demonstrated to be early sources of IL-12 in mice infected with *T. gondii *[26,27] and their importance for host protection against this parasite has been recently highlighted [28,29]. A similar protective role of these cell populations may also be important for host resistance against *N. caninum* infection. Our results indicate that such a protective immune response may be triggered already at the mucosal immune system in hosts challenged with this parasite in the GI tract. The IL-12 production detected in the MLN of the infected mice may contribute for the differentiation of IFN-γ-producing CD8^+^αβTCR^+^ IEL, found in higher proportions in these mice. Previous works have reported the importance of MLN [30], and of MLN DC in particular [31], in generating CD8^+^αβTCR^+^ IEL. Primed IEL have been shown to mediate protective immunity to oral *T. gondii* infection in adoptive cell transfer experiments [32,33]. Therefore it could be expected that these cells would have a similar role in *N. caninum*-infected mice.

In bovine neosporosis, the study of CD8^+^ T cells mainly addressed their possible participation in the immune response associated with foetal loss [34-36]. Nevertheless, CD8^+^ T cells have been extensively demonstrated to have a host protective role against parasitic infections [37], including neosporosis [34]. The production of IFN-γ by CD8^+^ T cells, which was also detected in calves experimentally infected with *N. caninum *[35], is an important mechanism in their host protective role against parasite infections [38-42]. It has been particularly shown that CD8^+^ T cells were the main early producers of IFN-γ in murine toxoplasmosis and were involved in resistance to acute primary infection [43]. Our results indicate that in murine i.g.-established neosporosis, CD8^+^ T cells are also early major producers of the protective cytokine IFN-γ. These cells were found elevated in the MLN of infected mice but also in the spleen. It would be interesting to determine whether these cells were locally activated in the spleen and identify the antigen-presenting cells responsible for this activation. Elevated proportions of IFN-γ-producing CD4^+^ T cells were detected later than CD8^+^ counterparts in the spleen and MLN of infected mice and not at all in the intestinal epithelium. A predominant response of CD8^+^IFN-γ^+^ IEL as compared to CD4^+^ counterparts has been also observed in mice orally infected with *T. gondii *[44]. Why CD8^+^ T cells apparently respond faster than CD4^+^ T cells in the gut mucosa and draining lymph nodes remains to be determined. A hypothesis worth to explore could be that *N. caninum* differentially affect the class I vs class II major histocompatibility complex antigen presentation pathways.

Production of IL-12 by MLN DC elicited in mice orally infected with *T. gondii* oocysts was shown to depend on bacterial translocation, promoted by the inflammatory reaction in the gut that followed the oocyst administration [45]. As no evidence of significant intestinal inflammation was found in the *N. caninum*-infected mice, IL-12 production may depend mostly on the parasitic antigens. Production of the pro-inflammatory cytokine IL-17 was associated with intestinal inflammatory pathology [46-48]. Our results, by showing that production of this cytokine was not detected in elevated proportions of IEL or MLN T cells, are thus in agreement with the lack of evident inflammation in the gut of the infected animals.

Control of microbial induced inflammation, including that caused by protozoans, largely depends on the action of Treg cells [49,50]. The observation reported here, by showing a high suppressive function of Treg obtained from *N. caninum*-infected mice may provide an additional explanation for the success of *N. caninum* in colonizing its natural hosts, where it can persist in a symptomless condition [51]. This highly suppressive function was more evident when antigenic instead of polyclonal stimulation was used in the Treg in vitro suppression assay of T cell proliferation. It is thus plausible that *N. caninum*, as demonstrated for other protozoan parasites [52], might manipulate natural Treg function in order to favour its persistence within the host. Interestingly, a recent report on persistent *Salmonella* infection showed that Treg suppressive potency decreased from the acute to the chronic phase, significantly affecting bacterial burden [53]. It would be worthwhile examining if the suppressive function of Treg later in *N. caninum* infection could be diminished when the acute phase of infection is overcome. Here, the immunosuppressive function was revealed by the inhibition of both in vitro T-cell proliferation and cytokine production. Curiously, no significant suppression of IFN-γ production was observed in co-cultures of Treg from the MLN of non-infected mice and MLN responder cells whereas the Treg spleen counterparts were highly suppressive. Particular environmental conditions of the MLN [54] might have conditioned both Treg and T conventional cells responsiveness, as may be suggested by the lower cytokine production of the MLN responder cells upon induction with anti-CD3 and irradiated APC, when compared with similarly stimulated spleen counterparts. As a decrease of spleen and MLN CD8^+^IFN-γ^+^ T cells proportions and numbers to underneath basal levels by day 7 of infection was observed, it would be interesting to determine whether it may reflect Treg function. In fact, other reports on apicomplexan parasite infections show that Treg, apart from suppressing CD4^+^ T cell proliferation and cytokine production, similarly affect CD8^+^ T cells [55,56]. It would be also interesting to assess whether such a high suppressive activity could be induced in mice infected by using other parasite administration routes. Moreover, it would be interesting to evaluate whether *N. caninum* infection would affect Treg suppressive activity along the gestational period and its influence in the cytokine environment, since higher levels of IFN-γ were detected in infected pregnant dams carrying live foetuses [57].

The suppressive activity of T regulatory cells may pose an additional difficulty to overcome infection by means of vaccination, as previous remarked [58]. As a preferential involvement of CD8^+^ T cells in the mucosal immune response to *N. caninum* was shown herein, the stimulation of parasite-specific effector and memory CD8^+^ T cell responses at mucosal sites may be a privileged target to achieve in vaccination against horizontally transmitted neosporosis.

In conclusion, intragastric infection of C57BL/6 mice with *N. caninum* tachyzoites preferentially activates mucosal and splenic CD8^+^ T cells, resulting in the production of the host protective cytokine IFN-γ. Nevertheless, the highly suppressive Treg present in the spleen of *N. caninum*-i.g.-infected mice may contribute to the establishment of a chronic infection.

## Competing interests

The authors declare that they have no competing interests.

## Authors’ contributions

AC and MV conducted and supervised the experiments, analysed the data, and wrote the manuscript. AF, LT and AR assisted in the experimental design and data analysis, and contributed to the interpretation of results and manuscript writing. PF, JD, AAC, and RC conducted the experiments, analysed data and contributed to the interpretation of results. JM participated in the experiments on Figures 1 and 2 and contributed to the analysis and interpretation of results therein. AR participated in data acquisition in the experiments involving mice. All authors read and approved the final manuscript.

## Supplementary Material

Additional file 1 **Detection of *N. caninum *in the intestinal tissue of mice infected by the i.g. route.** Representative images showing a *N. caninum* tachyzoite in the murine intestinal tissue **(a** and **b)**, 12 h upon i.g. infection, detected by immunohistochemistry. *N. caninum* tachyzoite (brown colour, denoted by arrow). The selected area in **(a)** is presented at higher magnification in **(b)**. Bar=100 μm. Results are representative of data from two independent experiments. Click here for file

Additional file 2 **Proportions of Treg within splenic and MLN CD4**^**+**^**CD25**^**+ **^**T cells.** Flow cytometry analysis of intracellular Foxp3 expression in splenic and MLN CD4^+^ T cells from C57BL/6 mice, 4 and 7 days after i.g. challenge with PBS or 5 × 10^7^ *N. caninum* tachyzoites (NcT), as indicated. **(a)** Gating of CD4^+^CD25^+^ and of CD4^+^CD25^-^ T cells. **(b)** Numbers within dot plots correspond to mean ± one SD of Treg (Foxp3^+^ cells) frequency within gated CD4^+^CD25^+^ T cell population. **(c)** Numbers within dot plots correspond to mean ± one SD of the frequency of CD4^+^CD25^-^ T cells expressing Foxp3, in the spleen of non-infected or infected mice, 7 days upon the parasitic challenge. In each panel, results are of a representative experiment out of at least three independent experiments (*n*=5 in each group). Statistical significance between groups in panel c is indicated (*P<0.05). No statistically significant differences were observed in the frequencies of Treg and Teff between control and infected mice. Click here for file
