# Supplementary figures and images for: Mucosal and systemic T cell response in mice intragastrically infected with Neospora caninum tachyzoites
Source: Vet Res. 2013 Aug 10;44(1):69. doi: 10.1186/1297-9716-44-69 (PMC3751650; doi:10.1186/1297-9716-44-69)

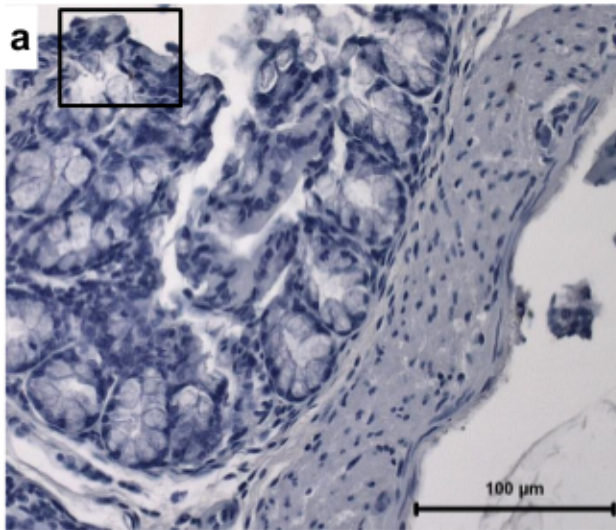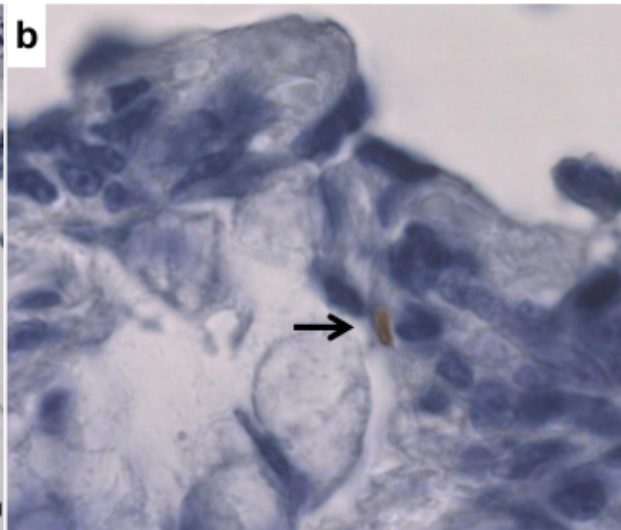

Supplement: Additional file 1 — Detection of N. caninum in the intestinal tissue of mice infected by the i.g. route. Representative images showing a N. caninum tachyzoite in the murine intestinal tissue (a and b), 12 h upon i.g. infection, detected by immunohistochemistry. N. caninum tachyzoite (brown colour, denoted by arrow). The selected area in (a) is presented at higher magnification in (b). Bar=100 μm. Results are representative of data from two independent experiments. [file 1297-9716-44-69-S1.pdf]

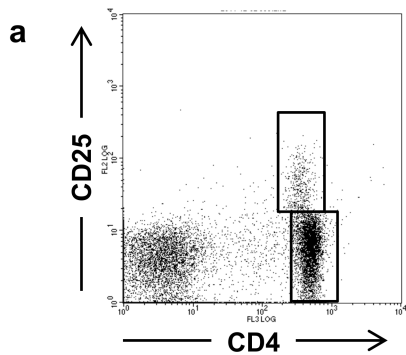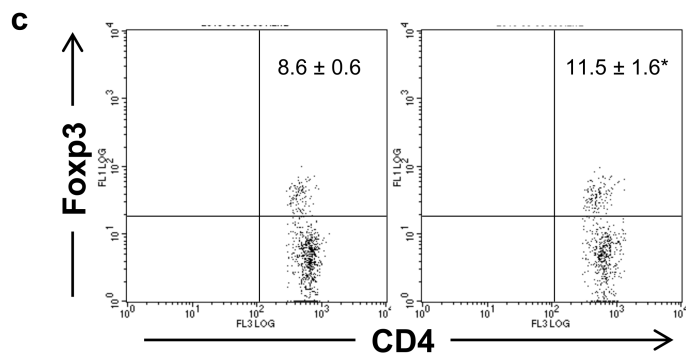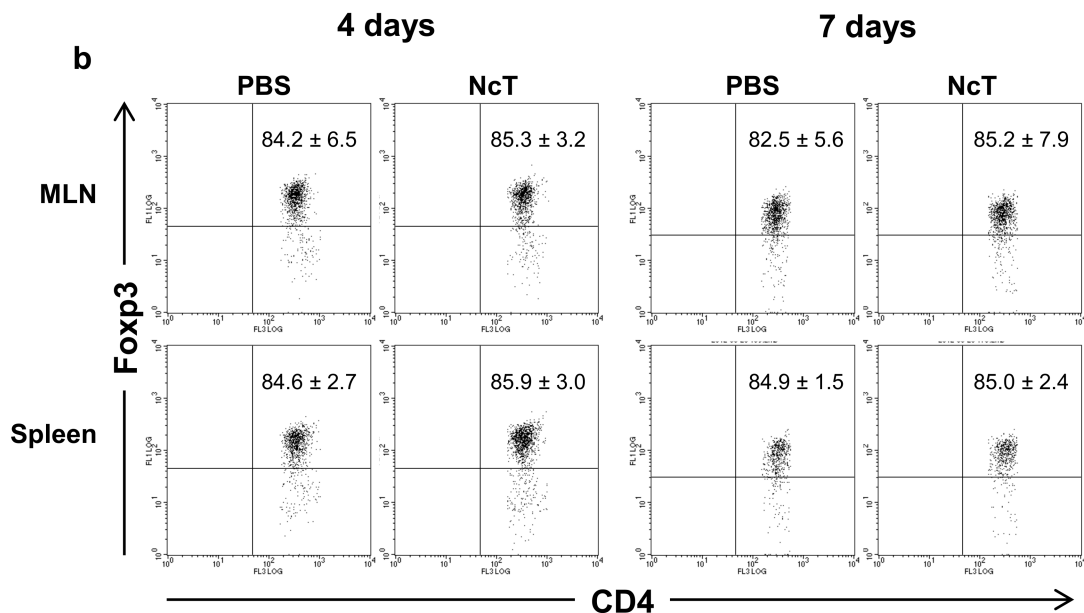

Supplement: Additional file 2 — Proportions of Treg within splenic and MLN CD4+CD25+ T cells. Flow cytometry analysis of intracellular Foxp3 expression in splenic and MLN CD4+ T cells from C57BL/6 mice, 4 and 7 days after i.g. challenge with PBS or 5 × 10^7^ N. caninum tachyzoites (NcT), as indicated. (a) Gating of CD4+CD25+ and of CD4+CD25- T cells. (b) Numbers within dot plots correspond to mean ± one SD of Treg (Foxp3+ cells) frequency within gated CD4+CD25+ T cell population. (c) Numbers within dot plots correspond to mean ± one SD of the frequency of CD4+CD25- T cells expressing Foxp3, in the spleen of non-infected or infected mice, 7 days upon the parasitic challenge. In each panel, results are of a representative experiment out of at least three independent experiments (n=5 in each group). Statistical significance between groups in panel c is indicated (*P<0.05). No statistically significant differences were observed in the frequencies of Treg and Teff between control and infected mice. [file 1297-9716-44-69-S2.pdf]
